# The historical decline of tobacco smoking among Australian physicians: 1964–1997

**DOI:** 10.1186/1617-9625-4-13

**Published:** 2008-12-29

**Authors:** Derek R Smith, Peter A Leggat

**Affiliations:** 1 WorkCover New South Wales Research Centre of Excellence, School of Health Sciences, Faculty of Health, University of Newcastle, Ourimbah, Australia; 2 Anton Breinl Centre for Public Health and Tropical Medicine, James Cook University, Townsville, Australia

## Abstract

**Background:**

Physicians occupy an important position as tobacco control exemplars and their own smoking habits are known to influence how effective they may be in such a role.

**Methods:**

A comprehensive review of all published manuscripts describing tobacco usage rates and tobacco control activities in the Australian medical profession between 1964 and 1997.

**Results:**

Some of the earliest surveys revealed that around one-quarter of Australian physicians were smoking in the mid twentieth century, a rate which rapidly declined in the 1970s and 1980s, with reductions beyond that achieved by the general population.

**Conclusion:**

Overall, our review suggests that not only do contemporary Australian physicians smoke at very low rates when compared internationally, but that an active professional community can also make a real difference to the lifestyle choices of its own members.

## Background

Tobacco control represents a key facet of public health. Worldwide, smoking is the second most common cause of death and the fourth most common risk factor for disease. If smoking trends continue at the current rate, tobacco will be causing approximately 10 million deaths per annum by the year 2020, with around 650 million fatalities overall [1]. Global tobacco control is therefore very important, a realization which led to the World Health Organization Framework Convention on Tobacco Control (WHO-FCTC), an evidence-based treaty focusing on demand reduction and tobacco supply issues from a global perspective [2]. At a local level, individual physicians and scientists have often been at the forefront of tobacco control, due to the now well-known links between smoking and ill-health, as well as the direct evidence of sick patients whom they treat in daily practice. Physicians themselves have also played a key role in tobacco research, with one of the earliest epidemiological studies linking tobacco smoking with adverse health outcomes being conducted among a group of British doctors [3]. So groundbreaking was the original investigation by Doll and colleagues that it was republished 50 years later [4] and remains a cornerstone of preventive medicine literature [5-7]. Being on the frontlines of primary health care, physicians have always occupied a key position in tobacco control and are often viewed as healthy lifestyle role models by their patients, their colleagues and the communities in which they live. As early as thirty years ago, it had been suggested that physicians would be more effective at persuading patients to quit smoking, if they themselves did not smoke [8].

Despite these facts, medical professionals have not always set a good example for patients with regard to smoking [9,10]. In the early to mid 20^th ^century for example, some tobacco companies even used physicians to help advertise their products [11,12]. The relative success of certain campaigns in the United States (US) and Japan was most likely due to the fact that smoking rates among local physicians were reasonably high at the time. In the US for example, some of the earliest epidemiological research found that around 40% of physicians were smoking in 1959 [8], although their smoking rate had dropped dramatically by the 1980s [13]. A large study conducted in 1965 [14] revealed that 68% of male physicians in Western Japan were current smokers. By the mid to late 20^th ^century however, physicians in many countries had begun to heed a growing body of scientific evidence linking smoking with ill health, and many active smokers among them had begun to quit [15]. As the medical profession was early to notice the dangers of tobacco use, the national smoking rate of physicians in the US, for example, fell dramatically during this time [16-18]. Tobacco use among their Japanese medical counterparts was also seen to decline last century [19], albeit at a less rapid rate than some of their Western counterparts. Regardless of what country they live in, the ongoing collection and interpretation of epidemiological data on smoking habits among physicians continues to serve at least two important functions relevant for tobacco control. Firstly, this kind of data can help predict how effective any potential anti-smoking campaigns in a given country may actually be [9]. That is, it would be difficult to convince the general public not to smoke if physicians continued to do so. Secondly, it allows policy makers to determine how 'mature' a country's smoking epidemic currently is, and thus, how soon the overall community prevalence rate might decline [9].

As a nation, Australia has set many positive examples in the field of tobacco control. It was one of the first countries to test the tar and nicotine content of cigarettes, one of the first democracies to ban all forms of tobacco advertising, one of the first nations to introduce explicit health warnings on cigarette packets, and one of the first countries to run large scale anti-smoking campaigns for the public [20]. Partly due to these restrictions, Australia has now become a relatively difficult region for tobacco industries to operate in, with a wide variety of anti-smoking laws and an aggressive and well-organized anti-smoking movement [20]. Australia's community smoking rate has been steadily declining in recent years [21] and is currently one of the lowest in the developed world [1]. Although part of the impetus for these changes can be attributed to the efforts of Australian physicians, few authors have systematically investigated tobacco smoking rates within this occupational subgroup [22]. The aim of our current review therefore, was to undertake a comprehensive and systematic review of tobacco smoking habits within the Australian medical profession during the mid to late 20^th ^century.

## Methods

An extensive literature review was conducted which targeted all manuscripts published in peer-reviewed journals relating to the topic of tobacco smoking among Australian physicians. As the official language of Australia is English, and studies from this region could be expected to be published in such a format, only English-language manuscripts were included. The review itself began with a search of relevant Medical Subject Headings (MeSH) such as 'smoking', 'Australia', 'tobacco' and 'physician' on the PubMed database from the US National Library of Medicine [23]. After identifying some initial studies, the search was repeated using keyword variations such as 'smoke' and 'doctor'. As there were relatively few older manuscripts on this topic listed in PubMed, the reference lists of journal papers located using our initial criteria were subsequently examined to find additional publications. Manuscripts were then arranged in descending order, depending on the year in which the smoking survey had actually been undertaken, rather than the year it was published. Where the year of study was not listed or was otherwise unclearly stated in the text, the manuscript's corresponding author was contacted for clarification, wherever possible. The publication year for all studies was eventually determined using either the manuscript itself or by direct contact with the authors. After sourcing the original article, all manuscripts were assigned a reference number based on the abovementioned criteria. For consistency, all smoking prevalence rates were rounded to the nearest whole number. Response rates for each study were also rounded to the nearest whole number for standardization purposes. To gain some perspective on physician's tobacco smoking habits over time, with respect to physicians in other countries and the general Australian population, smoking rates for these subgroups were also sourced from various scientific reports.

## Main findings and discussion

A total of 11 studies which investigated tobacco smoking habits among Australian physicians appear to have been published in the past 50 years [24-34], as shown in Table 1. All had been conducted as self-reporting postal surveys. The earliest study we located was from 1964 [24], with the most recent being conducted in 1997 [34]. Five investigations had sourced their participants from lists of registered medical practitioners [24-27,32], two had sent surveys to readers of their specific journal [28,29], two studies recruited physicians who were enrolled in postgraduate training programs [30,31], one study targeted physicians on a commercial mailing list [34], while the recruitment method of one other investigation was not clearly specified [33]. Response rates ranged from 14% [29] to 80% [27], with most above 50% [26,27,30-34]. Most studies listed the current prevalence of cigarette smoking among their physicians, although some earlier research suggested that a certain proportion also smoked pipes and cigars during the mid-twentieth century. This latter result was not entirely surprising, as Doll et al [35] has previously documented how a large proportion of their British physicians smoked pipes or cigars rather than cigarettes, similar to Fowler et al's [36] earlier finding in the same country. In contemporary Australian society however, pipe and cigar smoking is comparatively rare, with most active smokers consuming only cigarettes [37].

**Table 1:** Historical research on tobacco use among Australian physicians

**Authors**	**Year**^a^	**Participants and Recruitment Method**	**Number**	**Response**
Young & Ward [34]	1997	General practitioners who were on a commercial mailing list	311	73%
Young & Ward [33]	1996	General practitioners in Australia (selection method not specified)	855	67%
McCall et al [32]	1994	General practitioners registered with the Commonwealth Department of Human Services	318	59%
Roche et al [31]	1990	Physicians enrolled in a postgraduate family medicine training program	908	55%
Roche et al [30]	1991	Physicians enrolled in various postgraduate training programs	1361	55%
Nyman [29]	1989	Readers of the journal: *Australian Family Physician*	185	14%
Anonymous [28]	1982	Readers of the *Australian Medical Association Gazette*	1500	N/A^b^
Dodds et al [27]	1977	Physicians registered with the Medical Board of Victoria	275	80%
Rankin et al [26]	1974	Physicians listed in the Medical Directory of Australia (1972 Edition)	1276	69%
Anonymous [25]	1970	Physicians who were registered as medical practitioners in Australia	5708	40%
Anonymous [24]	1964	Physicians who were registered as medical practitioners in Australia	4348	33%

^a ^Year the study was undertaken, not the publication year, ^b ^The exact response rate was not stated for this article, only that 1500 questionnaires were mailed out

**Figure 1:**
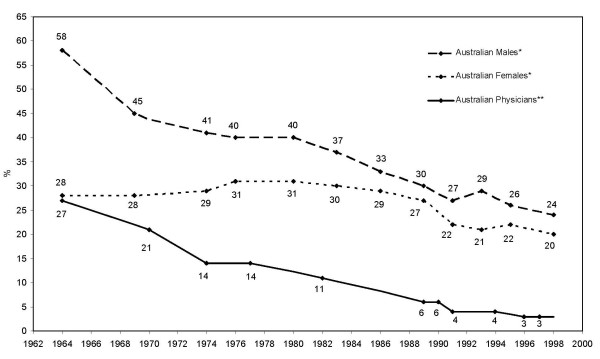
Historical comparison of cigarette smoking among Australian physicians and the general population (Data adapted from *References [37-44] and **References [24-34]).

The historical decline of tobacco of cigarette smoking among Australian physicians is displayed in Figure 1. In the early 1960s, research suggests that a large proportion of the general Australian population was consuming tobacco, with almost two-thirds of males and over one-third of females smoking cigarettes. Almost thirty percent of physicians were smoking in the 1960s, although their prevalence rate had declined dramatically by the 1970s, and was roughly half of what it had been by 1974 and 1978. By 1982 only about one-tenth of Australian physicians were still smoking, and this rate declined even further in later years. By the 1990s, only one-in-twenty Australian physicians reported themselves to be current smokers. On the other hand, tobacco usage rates among the general Australian population between 1962 and 1986 were less encouraging. Marked differences were found between the genders for example, with the male smoking rate being roughly halved during this period [37-44]. Female smoking rates in the general population increased slightly however, between the 1960s and the 1980s, before declining to about one-in-five by the 1990s. When compared to the general population, it can be seen that Australian physicians were only half as likely to be cigarette smokers in the 1960s, and only one-seventh as likely to be active tobacco smokers by the 1990s. The two most recent surveys that we located [33,34] suggest that smoking is now very rare within the Australian medical profession.

Even so, it is important to consider our results within the limitations of collected data, namely the fact that all studies relied on self-reported measures of smoking. As such, the possibility of systematic under-reporting of smoking habits by physicians cannot be totally excluded, and may have occurred for at least two reasons. Firstly, underreporting is a known confounder in population surveys that deal with personal habits, about which people may feel embarrassed. Secondly, and perhaps more importantly, is the fact that throughout the latter half of last century the increasing weight of scientific evidence had made it clear that physicians should not be smoking. While the overall community data no doubt suggests that fewer Australians are smoking than ever before, exactly how many of the physicians who claim to be 'non smokers' never use tobacco at all, cannot be established. As such, it is reasonable to assume that at least some physicians who smoked might have been reluctant to admit the fact during a survey. While we cannot be sure exactly what proportion of smoking doctors would incorrectly report themselves to be non-smokers, we anticipate it should be a relatively low proportion of the total. Furthermore, as this unique bias would presumably be occurring whenever physicians were surveyed in any country, international comparisons between them remain valid.

**Figure 2:**
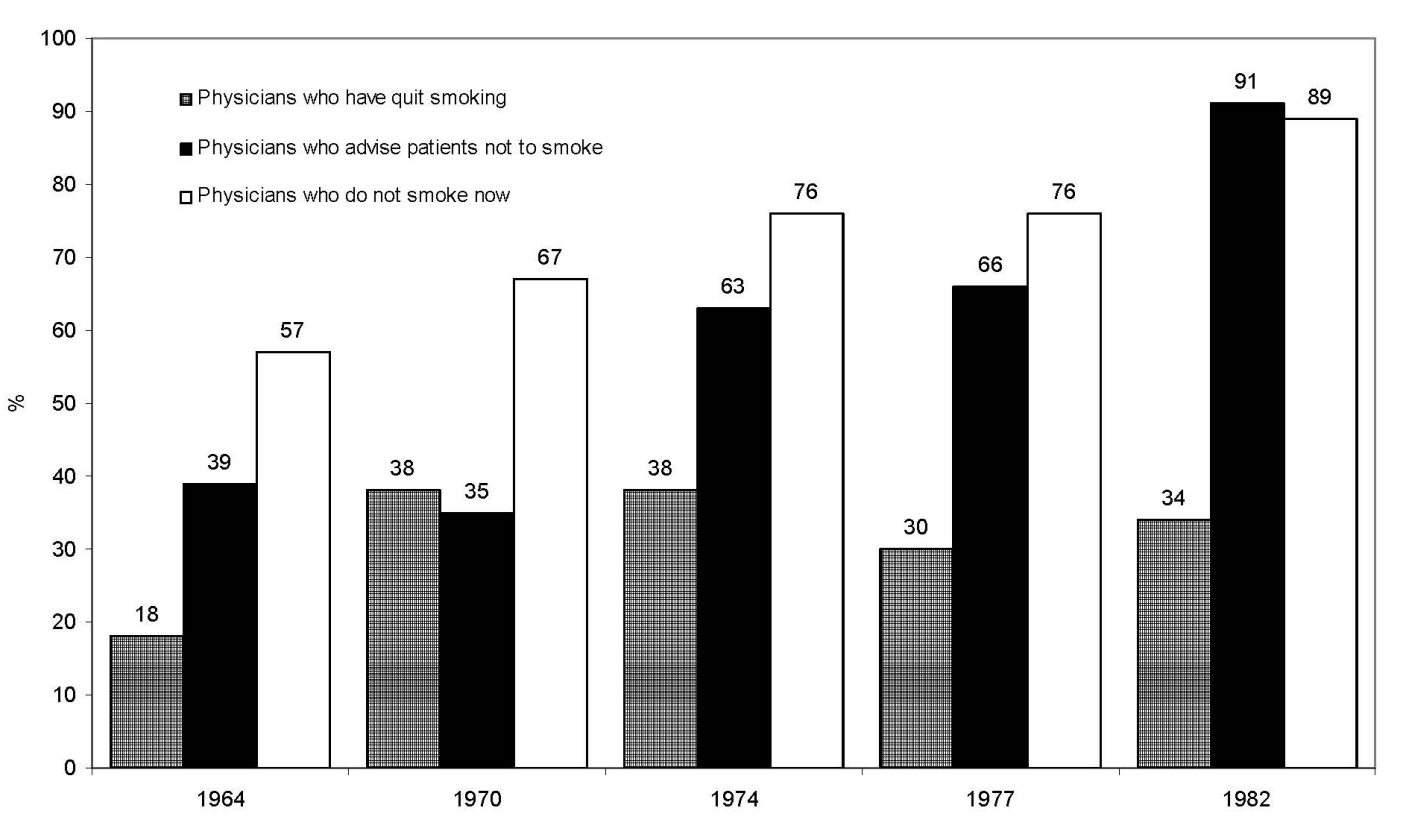
**Historical progression of non-smoking Australian physicians and their anti-smoking advice (Data adapted from References **[24-28]).

Methodological limitations notwithstanding, the proportion of Australian physicians who reported themselves as non-smokers or ex-smokers over the years is displayed historically in Figure 2. In 1964 over half were apparently non-smokers, a rate which had risen dramatically by 1982. The proportion of Australian physicians who had quit smoking also appeared to rise during this period, from 18% in 1964 to 38% between 1970 and 1974. By the late 1970s to early 1980s, around one-third reported themselves to be ex-smokers, although the increasing number of physicians who had never smoked was naturally reducing the overall proportion who could be ex-smokers. For these reasons, the proportion of ex-smokers remained relatively stable after 1970, as shown in Figure 2. The anti-smoking counseling behavior of Australian physicians also appeared to improve dramatically between 1964 and 1982. In 1964 for example [24], less than half of those surveyed (39%) were actively advising their patients not to smoke. By 1982 however, almost all physicians (91%) reported that they were counseling patients in this regard [28]. As with most countries, the intrinsic dangers of tobacco smoking had been almost universally accepted by Australian physicians by the mid-to-late 20^th ^century. Indeed, the two earliest studies identified during our investigation had actually asked their participants such a question. In 1964 for example, 96% of Australian physicians believed that cigarette smoking was a health hazard [24]. Encouragingly, the proportion of affirmative responses to this question had increased to 98% by 1970 [25]. These results suggest that physicians who continued to smoke after 1970 (over 10% of the group) were no doubt aware of the personal health hazards they faced.

Aside from changes to individual behavior, the Australian Medical Association (AMA) also became increasingly active in its opposition to tobacco usage throughout the 20^th ^century. Although many different reasons can be proposed as to why physicians would choose to be involved in the anti-smoking movement, it is reasonable to assume that physicians were probably the first health care providers to graphically witness tobacco induced diseases among patients in their care. Immediately following the Second World War for example, Gallup polls suggested that around three-quarters of all Australian adult males were current smokers [37]. This situation no doubt inspired physicians who were interested in preventive medicine, particularly as the scientific evidence against tobacco mounted. Changes of opinion in the Australian medical community were also reflected in the scientific evidence published in relevant Australian medical journals. In 1953 for example, the Medical Journal of Australia published its first article suggesting a tentative link between smoking and lung cancer [45]. In 1962, the National Health and Medical Research Council (NHMRC) issued an official statement on the suspected relationship between cigarette smoking and lung cancer [46]. Two years later in 1964, the first study on physicians' smoking habits was published in Modern Medicine of Australia [24], and three years after that, the AMA released their official policy on the topic [47]. By 1970, it appears that most of the Australian medical community had accepted the dangers of tobacco use [25].

Tobacco smoking research also appears to have begun on a national scale around this time, with the first national data on Australia's smoking demographic being published in the MJA during 1975 [38]. The Australian Bureau of Statistics (ABS) then conducted a national survey of alcohol and tobacco usage, publishing their results in a large report three years later, in 1978 [48]. Around this time, some Australian physicians became actively opposed to smoking at an individual level. By 1983 for example, at least four had been charged after defacing cigarette billboards with anti-smoking messages [49]. Australian health professions also adopted a more unified, national front, with nine of Australia's professional health bodies endorsing a national newspaper advertisement condemning cigarette promotion in 1987 [50]. In 1988, an article in the Medical Journal of Australia went one step further to suggest that a smoke-free Australia could be a bicentennial resolution for the year 2000 [51]. The introduction and approval of pharmaceutical products to help smokers quit their habit was instrumental in the fight against tobacco. In 1993, Nicotine patches were first approved for use in Australia, if prescribed by medical practitioners. From a policy perspective, the Australian medical profession became increasingly active in its opposition to smoking. In recent years for example, professional medical societies such as the Australian Medical Association [52], the Royal Australian College of General Practitioners [53] and the Royal Australasian College of Physicians [54] have all published their official policies on tobacco smoking and tobacco control. With regard to contemporary smoking interventions from a global perspective, the aforementioned WHO-FCTC [2] represents an important treaty and one to which Australia is a signatory.

Although few contemporary physicians appear to be smoking in Australia, it is interesting to compare the progression of their tobacco usage trends with physicians in other developed countries. Although the current paper is not intended as a systematic review of tobacco smoking in all countries, a few brief comparisons with other regions are appropriate. Some interesting contrasts can be seen when current tobacco smoking trends among physicians in four developed countries are juxtaposed with the five Australian studies during this time period [30-34]. Between 1990 and 2002 for example, at least five separate studies from the US [55-59] found that American physicians smoked tobacco at very low rates, similar to their Australian counterparts. From the publications we located, it appears that the prevalence rate of smoking among US physicians is currently between 2% and 4%, very similar to their Australian counterparts, where it ranges from 3% to 6%. Interestingly, three of the aforementioned US investigations also looked at smoking among dentists at the same time [55,56,59]. All three found that smoking was similarly rare among US dentists. These studies suggest therefore, that tobacco smoking is probably uncommon among medically-trained health care workers in the United States. As such, a summary of these studies suggests that fewer than one-in-ten American or Australian physicians, currently smokes tobacco. Physicians in both countries therefore, could be described as tobacco control exemplars, at least with regard to anti-smoking health promotion.

On the other hand, contemporary tobacco smoking rates among physicians in some other developed countries is less encouraging, with research among Italian doctors warranting particular concern [60]. Between 1995 and 2000 for example, five separate investigations by Italian researchers found that between 24% and 39% of physicians reported themselves to be active smokers [61-65]. From these studies it appears that Italian physicians may not be setting a good example as role models for their patients, at least as far as tobacco smoking is concerned. Somewhat disturbingly, La Vecchia et al [64] also reported that female Italian physicians may even be smoking at higher rates than the community in which they live. On the other hand, an Italian study by Nardini et al [63] found that while 39% of the physicians surveyed were current smokers, their rate was actually one of the lowest of all staff at a general hospital, particularly when compared to nursing students and auxiliaries. Future reductions of tobacco usage among Italian health care workers may therefore be difficult to achieve, although exactly how Italian physicians can and should move forward is beyond the scope of the current paper.

While a detailed discussion of smoking in Japan is beyond the scope of the current paper, some comparisons between the smoking habits of Australian physicians and their Japanese counterparts is still worthwhile. At least five English-language studies have looked at tobacco smoking among Japanese physicians in recent years [66-70]. The highest prevalence rate appears to have been documented in 1990, when 32% of Fukuoka physicians reported themselves to be smokers [66]. High smoking rates were also found among physicians during local surveys conducted in Tokyo [67], Okayama [68] and Fukui [69]. A nationwide survey of Japanese physicians during the year 2000 revealed that 27% of male physicians and 7% of female physicians smoked, a rate which was about half that of the national average for the general population, at the time [70]. Even so, from these results it can be seen that at least one-in-five Japanese physicians still consumes tobacco on a regular basis. The continued high prevalence of smoking among them is somewhat ironic, as it was a Japanese physician, Takeshi Hirayama, who published some of the first scientific evidence linking lung cancer to passive smoking [71]. Hirayama's work has withstood the test of time and has now become a classic paper in tobacco control [72]. Similar to Australia, the Japanese tobacco control environment did improve somewhat in the late 20^th ^century. In recent years, more anti-smoking measures have been introduced to Japan, particularly after 1998 when lung cancer rose to become the leading cause of death, ahead of stomach cancer [73]. Although this is clearly a step in the right direction, further efforts will still need to be made to encourage Japanese physicians and the medical students who precede them, to quit smoking [74,75].

In leading the fight against tobacco, it is important for physicians in all countries to recognize their unique role as tobacco control exemplars. One reason that Japanese physicians continue to smoke at high rates may be because they lack awareness of their status as public role models [70]. But physicians *do *occupy a very important role in preventive medicine, in all countries, both as health care providers and as public health exemplars. Physicians are not only responsible for smoking cessation treatment, but also for anti-smoking campaigns [76]. The impact of their preventive efforts should not be underestimated, as it is well-known that medical interventions can be effective in helping patients to quit smoking [77]. Aside from being a non-smoking role model in public, the physicians' clinic and hospital should also be a model of non-smoking behavior [78]. Similarly, medical and dental students need to be encouraged not to smoke [79,80].

Although smoking rates within the Australian medical profession have clearly declined in recent years, there remains some debate as to whether physicians are doing enough to stop their patients smoking [81]. It has been suggested that physicians are not always successful in recognizing which of their patients actually smoke, with a study of Australian general practitioners for example [82], finding that only 56% of current smokers were correctly identified as such. Additional work to help convince the Australian public of the important role that physicians play in tobacco control may also be appropriate in future. In a survey conducted in the early 1990s for example, 52% of people still believed that 'a lot of doctors smoke' [83]. Despite these potential caveats, smoking rates in the Australian medical profession have clearly declined, with one of the more recent surveys of hospital personnel for example [84] finding that staff in the 'medical' job category still had the lowest smoking rate of all (around 2%). Significant reductions in the smoking rates of physicians in some other countries has also been occurring, particularly in the US [13]. From a historical perspective America was the first country to introduce health warnings on cigarette packets in 1966 [85], and by later that decade, the US Public Health Service had began to publicly promote physicians as non-smoking role models. Community smoking rates subsequently declined, and at least part of the credit for these achievements could be given to physicians.

On the other hand, tobacco use remains problematic in Japanese society, an issue that will require additional government commitment to help reduce the current burden [76]. From a philosophical perspective it is difficult to understand why any physicians would smoke at all, given that they are well-placed to not only receive information about the adverse effects of smoking, but also to understand the science behind it and act accordingly. As such, it is important that this type of health information reaches physicians promptly, both via government networks and professional medical associations. Even so, up-to-date epidemiological data on the smoking habits of physicians remains important for two reasons. Firstly, it helps predict how effective anti-smoking campaigns may be. That is, it would be very difficult to convince the general public not to smoke if their physician role models continued to do so [9]. Secondly, it allows policy makers to determine how 'mature' a particular country's smoking epidemic currently is, and thus, how soon the community prevalence rate may be expected to decline. Historical data on physician's smoking rates is particularly useful, as it permits analysis of social trends regarding tobacco use, and also allows policy makers to see what control strategies were successful in the past, and what policies might be appropriate in the future.

## Conclusion

Overall, this review suggests that the prevalence of smoking among Australian physicians has fallen dramatically throughout the latter half of the 20^th ^century, with reductions beyond that achieved by the general population. While tobacco usage rates in the community have generally declined over time, credit should be given to the small number of physicians who encouraged their patients to quit, long before the health risks had been widely accepted by the general community. Such behavior has not been universal, however, with some international comparisons suggesting that while Australian trends are generally similar to those seen in the US, further effort is still required in countries such as Japan and Italy. While a detailed discussion of why physicians in some countries smoke at higher rates than others is beyond the scope of this paper, it nevertheless, highlights an important topic for future research in tobacco control. Whatever their geographical region, the fact that any physicians continue to smoke is unfortunate given their status as exemplars, and implies that further preventive efforts will need to be focused on personal health behaviors. It is important that smoking in the Australian medical profession continues its decline in future years, so that physicians in all countries can lead the way as tobacco control exemplars in the 21^st ^century.

## Competing interests

The authors declare that they have no competing interests.

## Authors' contributions

DS conceived the idea for the study. DS and PL wrote the manuscript. Both authors read and approved the final version of the manuscript.
